# Leadless Pacemakers in Complex Congenital Heart Disease

**DOI:** 10.3390/jcm14238560

**Published:** 2025-12-02

**Authors:** Archana Rao, Elen Hughes, Milos Prica, Sadaf Raza, Mohammed Saber, Reza Ashrafi

**Affiliations:** Liverpool Heart and Chest Hospital, Thomas Drive, Liverpool L14 3PE, UK

**Keywords:** congenital heart disease, leadless pacemaker, shunt, Fontan, endocarditis

## Abstract

Pacing in complex congenital heart disease can be difficult and with significant drawbacks due to issues with infection and long-term leads within the vasculature. Leadless pacemakers have emerged as a new technology with a strong safety and efficacy record in normal cardiac anatomy. Here, we review the current available technology, current evidence in complex congenital pacing and how leadless pacemakers may be used in complex congenital heart disease.

## 1. Introduction

### Epidemiology

Complex congenital heart disease (CHD) is thought to occur in roughly 6/1000 live births [1] with a roughly stable incidence of CHD and complex CHD over recent years [2].

Whilst the incidence of congenital heart disease and complex congenital heart disease appears stable at birth, there has been a marked improvement in survival (in many studies) leading to an overall increased burden of CHD [3] and an older population at time of death [4].

As the population ages, the requirement for pacing is likely to increase over time for a variety of reasons such as repeated interventions, age-related fibrosis or as adjunctive management for arrhythmias. In our institution, as an adjunctive treatment, pacing support is used to facilitate drug treatment of patients with recurrent atrial arrhythmias and sinus node dysfunction. The exact prevalence of pacing is not fully understood in the complex CHD cohort due to often small groups across multiple institutions and gaps in data quality, but as an example, 7% of adults followed up in one large tertiary unit were found to have a pacemaker [5]; this is balanced against a population-wide prevalence of 0.4 per 1000 people aged 18–64 [6].

The specific influence of age on the requirement for pacing can be difficult to extrapolate as operative or interventional complications may cause a need for pacing in neonates, [7] but the median age in years for device implantation in congenital structural heart disease has been reported in a large study as 26 [8].

Epicardial pacing in complex CHD has been used for a very long time, and an atrial lead failure rate of 28% and a ventricular failure rate of 40% at 10 yrs have been reported [9]. This is used as a ballpark for many units when talking to patients. This compares to a much lower rate of lead failure in non-congenital cardiac surgery which has been reported to be as low as 1.6% for ventricular leads at 5 years [10]. These figures are often highly influenced by the patient cohort and implantation in very small children/neonates is particularly associated with poor lead survival [11]. Therefore, discussing the patient’s clinical background is particularly important to advise them correctly.

Survival of transvenous leads seems comparatively better than epicardial leads though with a higher rate of reinterventions for other reasons [12].

Broadly speaking, the existing outcomes for pacing in complex CHD show a significant burden of complications including high levels of lead revisions and infection [13] and occur at a greater rate than pacing in the absence of congenital heart disease.

Patients with congenital heart disease and transvenous pacing leads experience a much higher rate of lead failure compared to standard populations [14,15]. This may be due to somatic growth, complex diagnoses and intraprocedural variabilities [14].

## 2. Leadless Pacing

### 2.1. History of Leadless of Pacemakers

LPMs have been commercially available since approximately 2014. After over a decade of research, real-world data suggest robust safety outcomes [16] and offer insight into the management of devices at end of service [17].

Whilst there exists significant amount of outcome data in non-congenital heart disease patients, there is very little systematic data on LPMs on patients with complex congenital heart disease, and much of the data is limited to promising case series [18,19] or paediatric series [20].

An early collection of the summarised data in CHD including complex and non-complex cases totalled less than 30 patients but showed no complications and effective pacing [21].

### 2.2. Rationale for Leadless Pacemakers

Leadless pacemakers (LPMs) have become popular for a variety of reasons but the very low complication rates in large trials [22,23] and the extremely low infection rates when compared to traditional pacing systems [24] are particularly notable.

Beyond this, the ability to deliver pacing in difficult situations, such as patients on long-term dialysis, those with reduced vascular access, and young people for whom a desire to avoid a lead-based system is important, has furthered the uptake of LPMs among cardiologists.

LPMs may be particularly attractive in complex CHD for the following reasons:

1. Avoidance of atrio-ventricular valve interference;
2. No long-term vascular dwell;
3. No baffle obstruction;
4. Potentially psychologically (cosmetically) better due to elimination of generators [25].

### 2.3. Leadless Pacemakers

#### 2.3.1. Current Options

In current clinical practice, there are LPMs to cover most clinical scenarios from a variety of manufacturers. Briefly summarised in Table 1 [21,26,27,28,29,30,31] are the options currently commercially available or close to approval and in routine clinical practice. The list is likely to expand as leadless conduction system pacing develops [32].

Briefly, the device with longest commercial availability is the Medtronic Micra™ (Medtronic, Minneapolis, MN, USA) system which is available as a ventricular-only system either as a VVI system or as an atrio-ventricular synchronous system which senses mechanical atrial activity to provide VDD pacing.

The first LPM system was the Nanostim™ from Abbott™ (Abbott, Chicago, IL, USA), but this was recalled from clinical use due to battery issues and replaced with the Aveir™ system. The Aveir™ is available as standalone atrial or ventricular systems or with wireless communication between the two devices to provide dual-chamber pacing.

Recently, the Empower™ system (Boston Scientific, Marlborough, MA, USA) has been developed, which allows for leadless VVIR pacing with wireless communication to a subcutaneous defibrillator system to provide anti-tachycardia pacing guided by the defibrillator system. Research has recently been completed for this and an application for regulatory approval is expected soon [33].

The final leadless pacing system is the EBRwise™ system (EBR Systems Inc., Sunnyvale, CA, USA) which consists of an electrode placed directly in the left ventricle, which relies on a co-implanted right ventricular device to provide a pacing stimulus, after which it will provide a pacing stimulus to cause biventricular pacing. The leadless electrode is powered by piezoelectric crystals which receive ultrasound stimulation from a transmitter under the skin.

#### 2.3.2. Access Considerations

**Figure 1:**
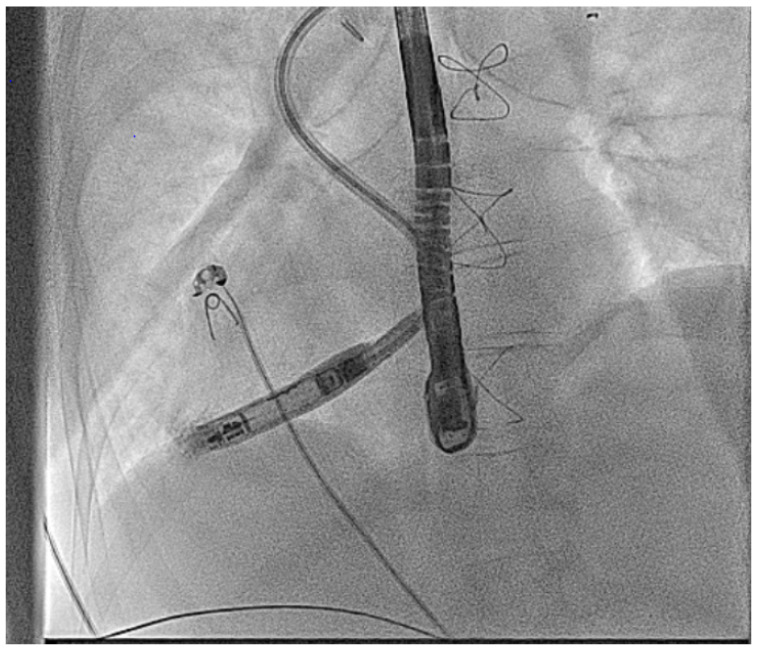
Access from the right internal jugular vein with the Aveir™ LPM in an adult patient with dextrocardia and interrupted IVC.

In addition to the intracardiac pathology, patients with complex CHD may have issues with access either from vascular occlusions due to multiple childhood procedures [34] or vascular abnormalities such as an interrupted inferior vena-cava (IVC) as seen in Figure 1 [35].

Most leadless pacemakers require large-bore access, commonly 23Fr and above depending on the manufacturer, and the use of vascular ultrasound is recommended when performing these procedures [36].

Pre-procedural imaging may be useful to understand vascular access and the optimal route for LPMs, whether the femoral or jugular approach is targeted, and assess the likelihood of successful delivery.

In the most complex vascular anatomies or where there is stenosis, adjunctive techniques such as stenting [37] or more complex wire-support techniques [38] may be required to overcome the challenges.

In addition to these complex vascular approaches, there have been case reports of surgical epicardial insertion of leadless devices [39], the use of leadless devices in small neonates [40], and implantation via a retrograde arterial approach [41].

#### 2.3.3. Anticoagulation

In most cases, whilst there is no consensus between manufacturers, operators will give intravenous heparin normally as a bolus dose of 5000 units. This dose may be revised dependent on body weight. In the long term, anticoagulation is not mandatory for leadless devices as is the case with transvenous devices, and it is thought that the parylene coating (a thin polymer coating that serves as a moisture and dielectric barrier for various components in electronics, medical and instrumentation industries) here maybe particularly less thrombogenic [42]. In cases of patients with significant right to left shunting or at thrombotic risk, anticoagulation is reasonable on a case-by-case basis.

#### 2.3.4. Vessel Closure

There is no current mandated/recommended mechanism of vessel closure for LPMs, and centres have used manual pressure, purse string or z-stitch techniques as well as vascular closure devices [43]. No clear benefit has been shown between different techniques for LPMs and much of the data is taken from other large-bore venous access procedures [44] particularly in electrophysiology.

Closure choice is likely to be based on the institution’s familiarity with differing techniques, bed availability pressures and cost.

#### 2.3.5. Retrieval Considerations

Whilst in a lot of cases published to date, retrieval has not been a major consideration, there will come a time where retrieval is either desired or required due to embolisation/infection or for another reason. The more complex the anatomy, the harder this may become and, to date, there is not a lot of data on long-term retrievability in congenital heart disease. At the moment, the most data comes from Nanostim™ with data up to 9 years with good outcomes [45] in a mixed cohort.

In structural CHD, there are no major cases series or significant case reports of device retrieval and, therefore, recommendations are not readily available. Operators will need to carefully consider some of the challenges they may face in structural CHD:

1. Vascular anomalies precluding easy femoral access;
2. Multiple abandoned leads causing obstruction;
3. Valve replacements;
4. Significant calcification;
5. Significant anatomical challenges such as very small or rotated sub-pulmonary ventricles such as in Ebstein’s anomaly.

**Figure 2:**
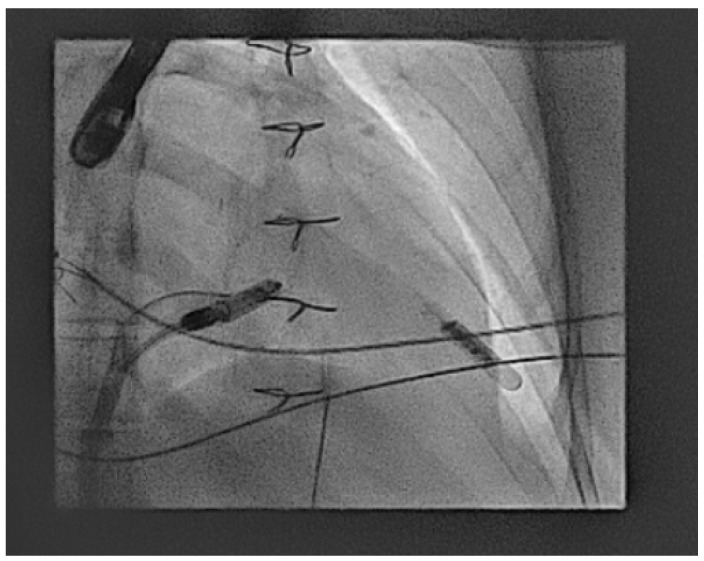
Extraction of an AV Micra™ due to poor AV synchrony in an adult patient with Ebstein’s anomaly and a tricuspid valve replacement using a dedicated retrieval catheter/system.

Shown below is a case from an adult patient with operated Ebstein’s anomaly with a tissue tricuspid valve replacement which required careful movement of the retrieval sheath across the valve replacement. The device was then fully brought in to a covered sheath to prevent the tines catching on the valve replacement (Figure 2 and Supplementary Video S1).

**Figure 3:**
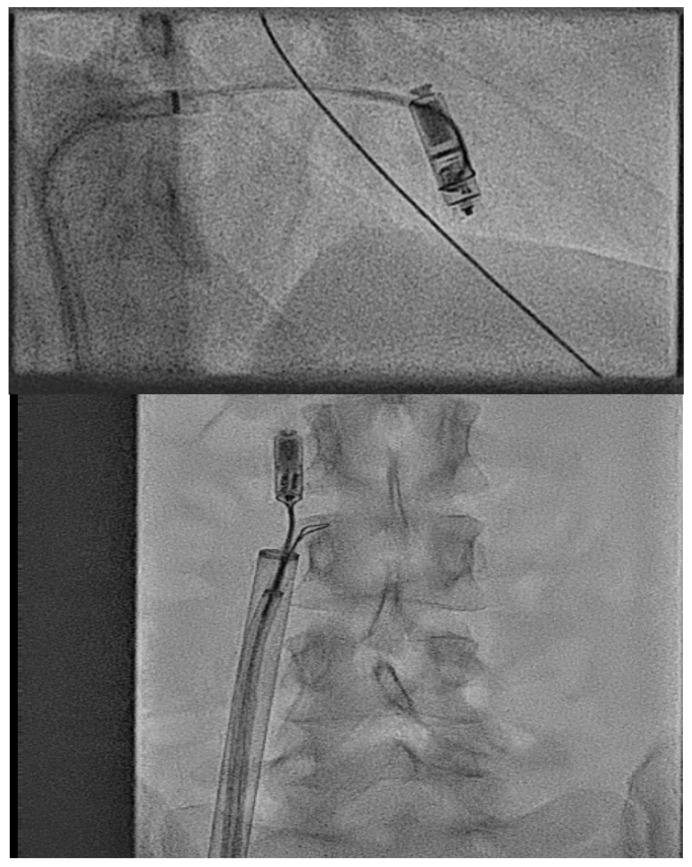
Acute explant of an AV Micra™ due to poor parameters using a goose neck snare and then reorientation of the device in the IVC to change to a more favourable angle to allow final removal.

In the next case, a Micra™ was released in an adult patient with congenitally corrected transposition of the great arteries (CCTGA), but after release, the immediate parameters deteriorated significantly. Here, a goose neck snare is used with a steerable sheath to grab the device back into the IVC and then from here the device is released and resnared to orientate the device successfully into the large sheath (Figure 3).

**Figure 4:**
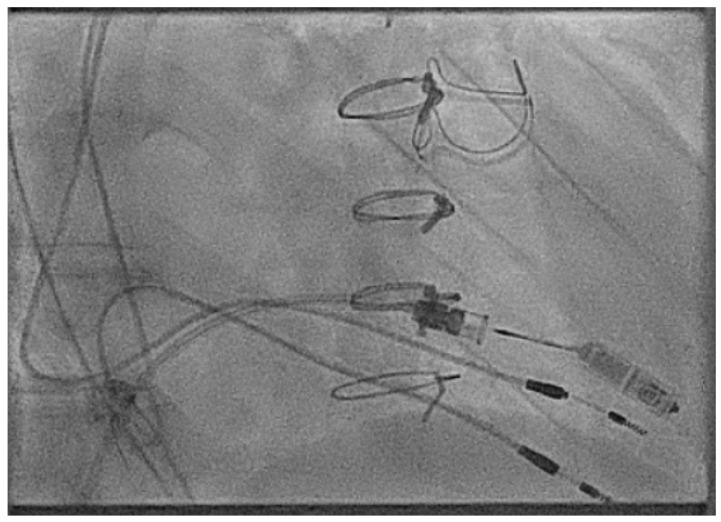
Extraction of a chronically implanted AV Micra™ due to poor parameters using the Abbott™ retrieval catheter to get past the small tricuspid annulus and multiple abandoned leads.

In this final retrieval case, an adult patient with repaired Tetralogy of Fallot (TOF) and multiple abandoned leads had a chronically implanted Micra™ removed due to rising threshold and to allow for more space long-term within the right ventricle. We suspect that the failure of the Micra™ was due to anatomical challenges made more difficult due to the passive tines. Here, there was little space through the tricuspid valve, and so using the retrieval catheter, the device is captured in the ventricle and then docked to allow enough support to push the protective sleeve over (Figure 4).

#### 2.3.6. Complications

The major differences, when compared to transvenous pacing in terms of complications, are the significantly higher risk of major tamponade [46], device embolization and access site complications [47].

Complications of LPMs in congenital heart disease are almost identical to those in structurally normal hearts with quoted complication rates of 0.5% [48]. One potential difference between the normal heart and CHD group may be a possibly lower tamponade rate because patients with complex CHD often will have had multiple sternotomies resulting in the development of pericardial adhesions [49].

#### 2.3.7. Atrial Leadless Pacemakers in CHD

Large-volume leadless atrial pacing has only begun in the last few years with the introduction of the atrial Aveir™ device, which is licensed for atrial only or as part of a dual-chamber system. The device itself is designed for implantation in the right atrial appendage using an active fix helix and the standard delivery catheter system.

In complex CHD, the atrial anatomy, atrial myocardial electrical quality and access to the atrium itself may all be compromised by the birth anatomy or subsequent interventions. Therefore, pacing can be difficult. Additional structural issues in CHD include the existence of shunts at the atrial level which risks systemic embolisation [50] and inadvertent deployment in the systemic circulation as it is not always easy to tell from fluoroscopic images where a device is located. In difficult anatomy where there is uncertainty, it can be useful to have contemporaneous imaging support such as intracardiac echocardiography.

**Figure 5:**
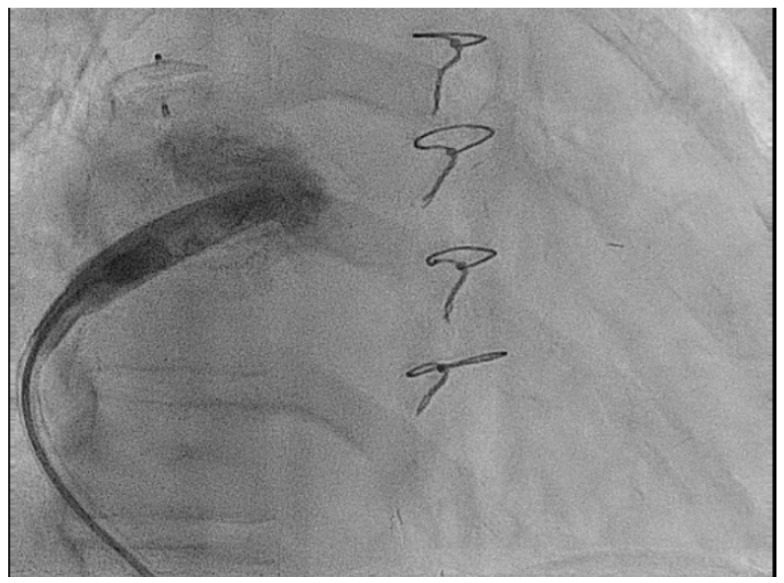
Contrast shot of the right atrial appendage in an adult patient with partially palliated tricuspid atresia and univentricular anatomy showing a very short appendage.

Prior to the development of the Aveir™ system, leadless atrial pacing was limited to case reports of off-license use of the Micra™ system in the atrial appendage and was reliant on suitable anatomy [51,52]. With the availability of the Aveir™ system, the options in terms of deployment have significantly increased but challenges do still exist and pre-procedural imaging/electroanatomical mapping can be particularly helpful in planning the device deployment (Figure 5).

**Figure 6:**
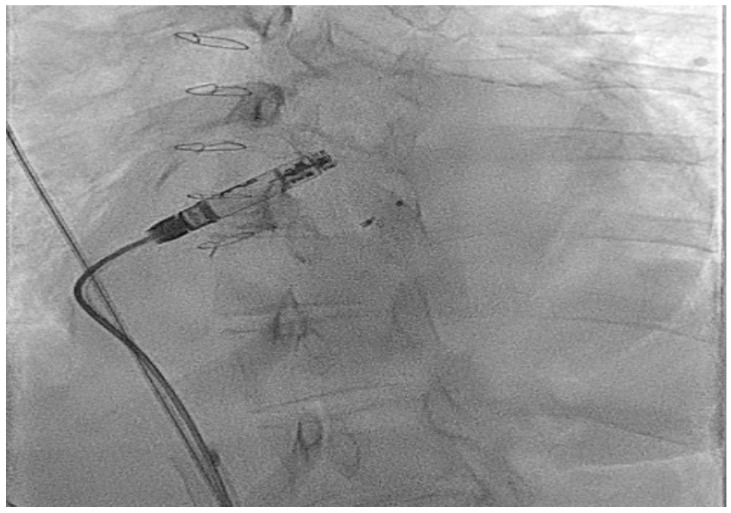
An atrial Aveir™ deployed to an area of healthy septal tissue in an adult patient with an atrio-pulmonary Fontan and sinus node dysfunction after identification of a suitable site at a previous electrophysiology procedure. The small target area was due to large areas of electrical scar.

A particular area of difficulty exists in the Fontan cohort especially when palliated with an atrio-pulmonary connection, and the atrial appendage is utilised to create the superior pathway to the pulmonary arteries. In this case, there will not normally be an appendage to anchor the device and another site will need to be utilised, which is not ideal as the embolisation risk is higher when the atrial device is deployed to a non-ostial appendage site [53]. In addition to the structural challenge here, many patients with an atrio-pulmonary Fontan will have suffered with arrhythmias and may have undergone extensive ablation therapy limiting target sites for deployment (Figure 6).

Another area of potential challenge specifically occurs in patients with an atrial switch procedure for transposition of the great arteries where the baffle pathway may make catheter delivery hard and where appendage delivery will be to a morphological left atrial appendage. In this case, as the phrenic nerve can sometimes be captured by pacing in and around this area, the operator will need to check carefully prior to device release.

**Figure 7:**
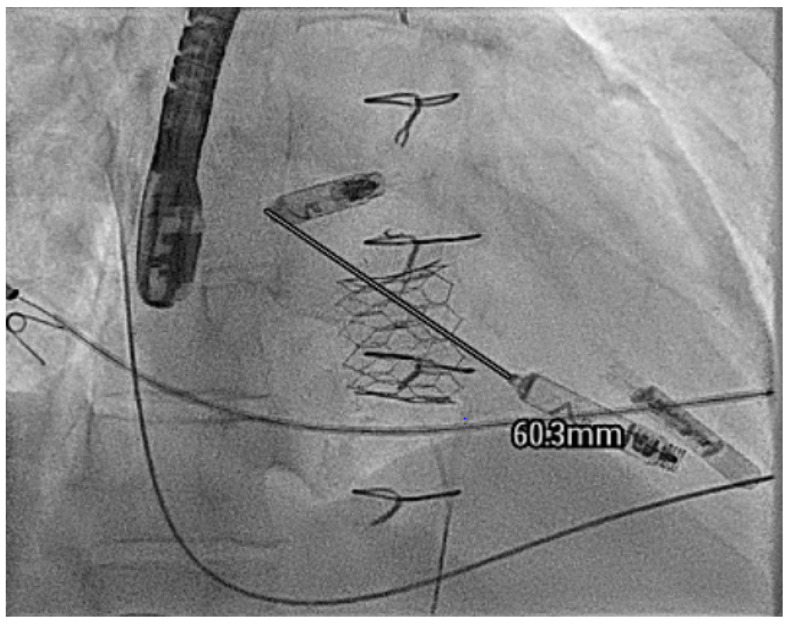
Dual-chamber Aveir™ system in place in an adult patient with Ebstein’s and huge right atrial dilatation with the distance between devices shown.

The Aveir™ system in its dual-chamber configuration communicates wirelessly between the two devices to provide AV synchrony. The exact amount of battery usage for wireless communication is affected by device-to-device orientation and the distance between the devices [54]. Wireless communication and battery consumption may be an issue in patients with very large right atria or extreme rotation due to volume overload or underlying pathology (Figure 7).

In these cases, there may be a need to inform the patient that a higher battery drain may be encountered and therefore more procedures over a lifetime.

#### 2.3.8. Ventricular Leadless Pacemakers in CHD

Ventricular leadless pacing in CHD has a much larger evidence base than its atrial counterpart, having been available as both the Micra™ and Aveir™ systems for much longer. Like the atrial device challenges, there will be issues with the ventricular device due to anatomy, myocardial electrical quality and access to the ventricle itself, which may all be compromised by the birth anatomy or subsequent interventions.

Typical issues that operators may face in complex congenital cases for ventricular pacing include a smaller ventricular cavity in Ebstein’s, large areas of septal scar in TOF, smooth sub-pulmonary left ventricles in CCTGA, atrio-ventricular (AV) valve replacements/stenosis or no access such as in an extra-cardiac Fontanconduit (ECC).

**Figure 8:**
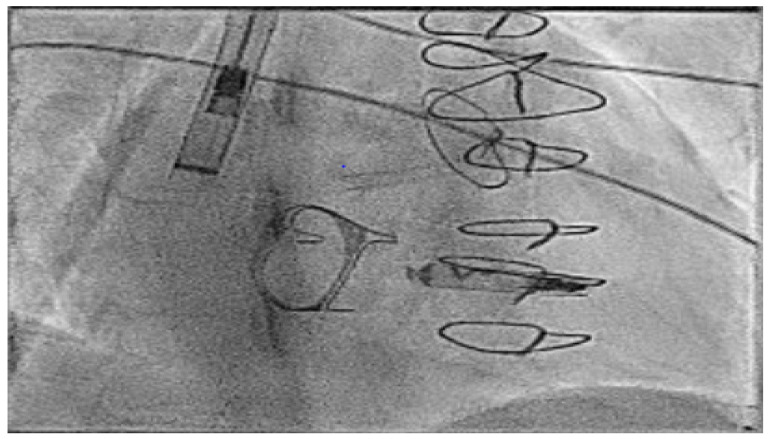
A VR Aveir™ in an adult patient with a very small right ventricular cavity and the device deployed as far from the AV valve replacement as possible but still very close.

In pathology with small ventricular cavities, there is often not much that can be achieved from a positioning perspective, and the main goal will be to clear the AV valve to maintain device stability; however that can be achieved. Sometimes this may necessitate a position towards the outflow tract or more apical position than may initially be desired (Figure 8).

**Figure 9:**
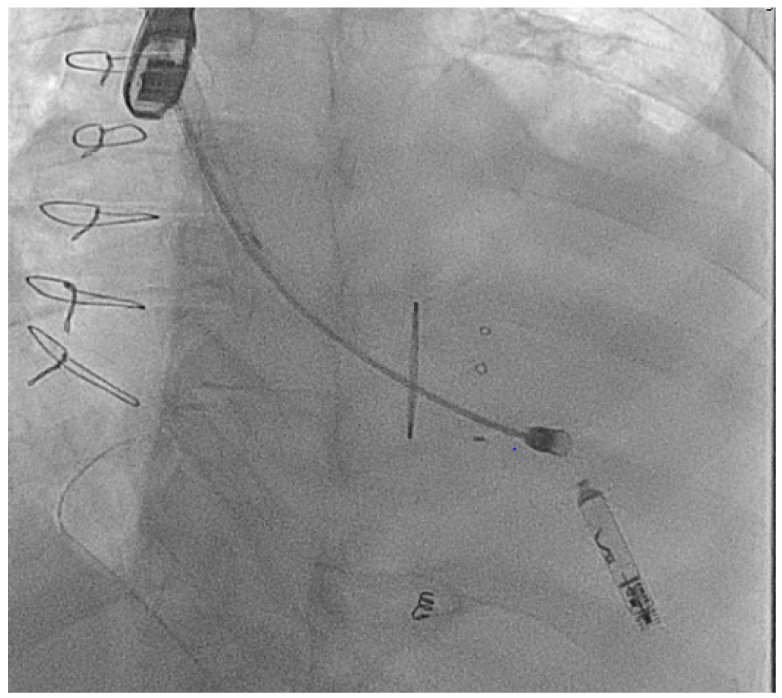
A VR Aveir™ delivered from the right jugular vein across a stenotic AV valve replacement in an older adult patient with Ebstein’s.

In patients with AV valve stenosis or replacements, it can be very difficult to cross the valve directly with the device/delivery catheter and in these cases, it can be necessary to utilise other techniques to help cross the valve. The first manoeuvre we would suggest would be to change the access point and the jugular approach may change the trajectory enough to make it easier to cross the valve (Figure 9).

When the change in angle has proved insufficient to overcome a difficult to cross AV valve, we suggest utilising techniques from structural valve interventions to help.

**Figure 10:**
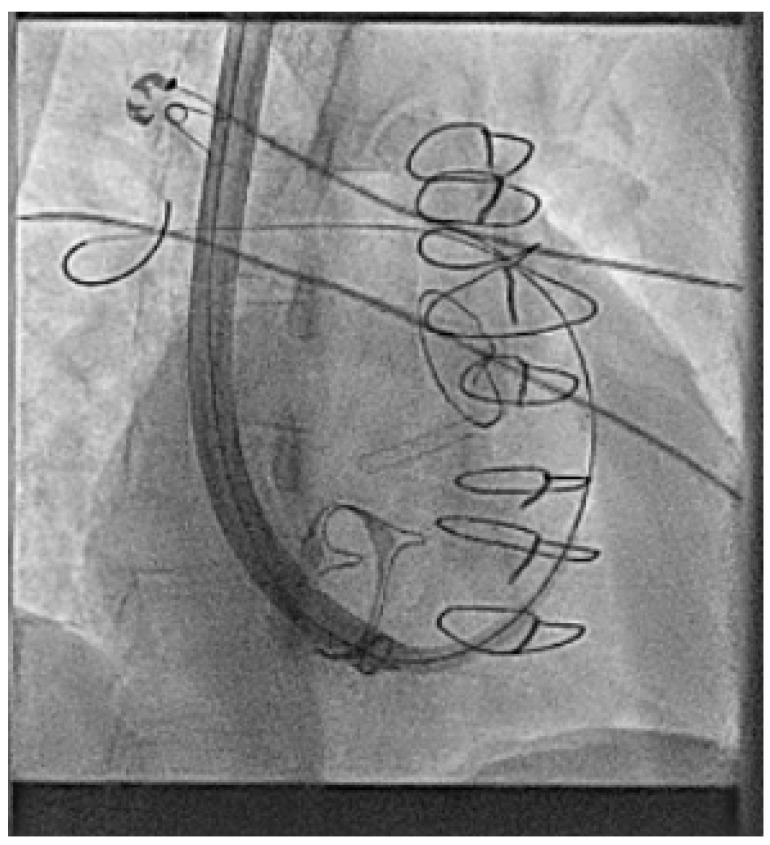
A Gore™ DrySeal taken over a stiff wire direct to the ventricle to allow for deployment of a LPM in an adult patient with tricuspid valve replacement stenosis.

In these cases, the valve can be first crossed with a diagnostic catheter and then a stiff structural support wire such as Lunderquist™ (Cook medical, Bloomington, IN, USA) deployed in the distal pulmonary artery bed. Then, a large-bore flexible structural sheath such as a Gore™ DrySeal (W.L. Gore & Associates, Inc., Flagstaff, AZ, USA) can be taken with a dilator over the valve and into the ventricle directly. The delivery catheter can then be uncovered directly by pulling the delivery sheath back across the AV valve and keeping the catheter in the ventricle, thus overcoming the difficulty with the AV valve (Figure 10 and Supplementary Video S2).

**Figure 11:**
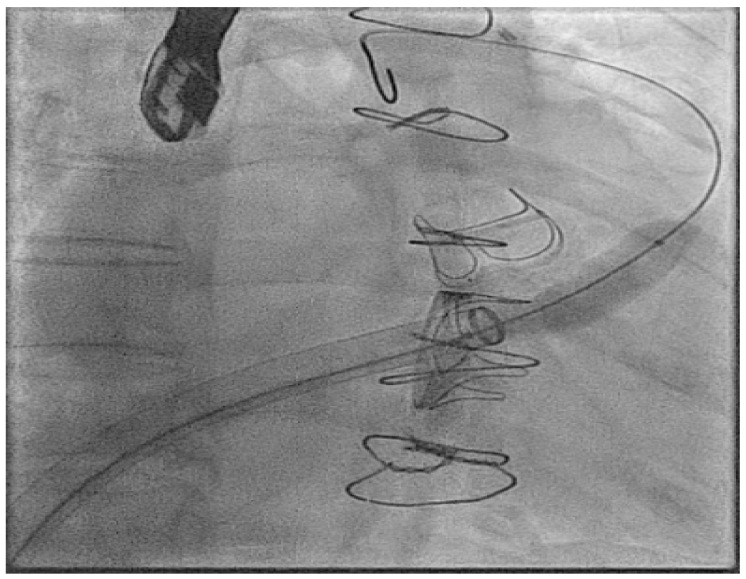
A Gore™ DrySeal taken over a stiff wire direct to the ventricle but with a structural sizing balloon used as an anchor to push the sheath across a stenotic valve replacement where the dilator had failed to support sheath movement. The patient was an older adult patient with significant calcific valve stenosis.

Occasionally, a combination of valvular stenosis and difficult angle may make these techniques insufficient in complex CHD and a lack of support to cross the AV valve may occur. A final option we have utilised is to use a structural sizing balloon as an anchor to push distally after the valve annulus to allow thesheath to come over stenotic valve leaflets (Figure 11).

In patients with sub-pulmonary ventricles that are of left ventricular morphology, there may be a lack of classical trabeculations and the endocardial wall may be smoother than in a normal right ventricle, which can make deployment of tine-based LPMs more difficult. In these cases, we would normally suggest that an active fixation device may be better from a stability perspective.

The most complex challenge in leadless ventricular pacing is probably in the cohort of patients with a Fontan circulation. These patients will be very unlikely to have easy access to paceable ventricular tissue.

**Figure 12:**
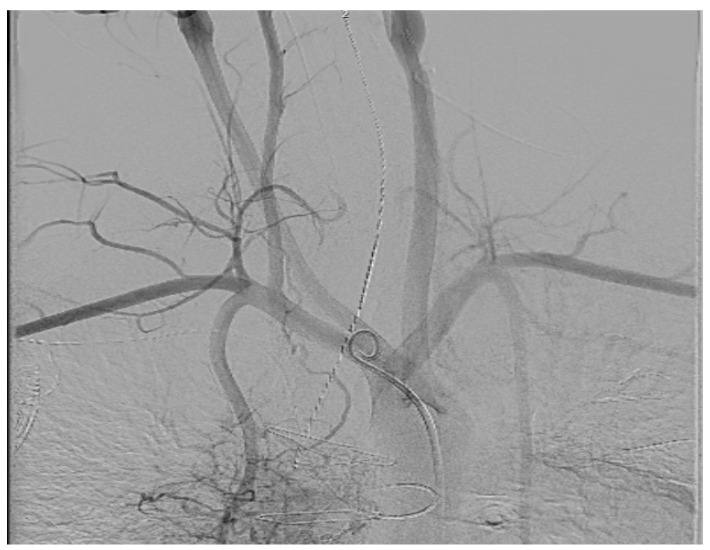
A digital subtraction angiogram after removal of the 26 French introducer sheath from the left carotid showing no complications in an adult patient with retro-aortic LPM insertion.

Historically, the majority of pacing for these patients has been epicardial with a small amount of transvenous pacing either via the coronary sinus or complex hybrid procedures [55]. A worry with these procedures has, in particular, been the risk of embolic phenomena due to leads in the systemic circulation [56]. The ability of the leadless pacemaker to sit isolated within the ventricle with a parylene coating has made operators consider whether they may be less of a thrombotic risk and therefore an alternative to high-risk epicardial surgical procedures. Delivery of the large-bore sheath has been the major barrier to units considering this approach for patients, but again extrapolating from other structural valvular techniques, we have been able to find novel ways to perform this procedure safely. The first option we have used is built on the carotid access used in transcatheter aortic valve replacement [57] and similarly uses a direct surgical cutdown to deliver the access sheath without complication (Figure 12).

Whilst the carotid offers a straight and easy insertion route for ventricular devices, this may not always be attractive depending on the availability of vascular surgical support or due to the risk of vascular thrombotic complications in older patients. To overcome this, we developed a transbaffle approach to increase the number of patients with a Fontan circulation who could be offered LPMs [58].

**Figure 13:**
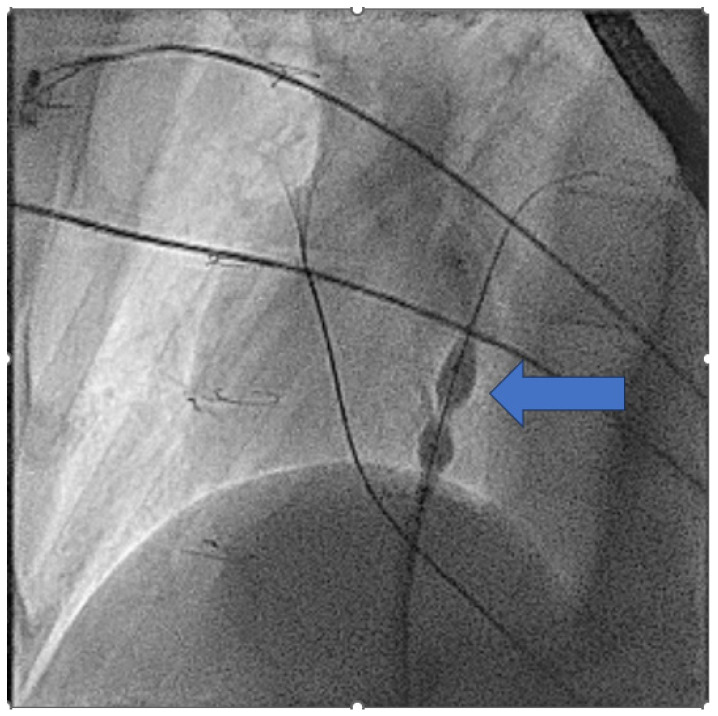
Enlargement of the transbaffle puncture in a patient with an ECC using an 8 mm non-compliant structural balloon (arrowed).

In these cases, normally performed under general anaesthesia, a transbaffle puncture is performed with a radiofrequency needle to ensure a clean puncture. The puncture site can then be enlarged, normally to an 8 mm size, with a non-compliant structural balloon (Figure 13 and Supplementary Video S3).

**Figure 14:**
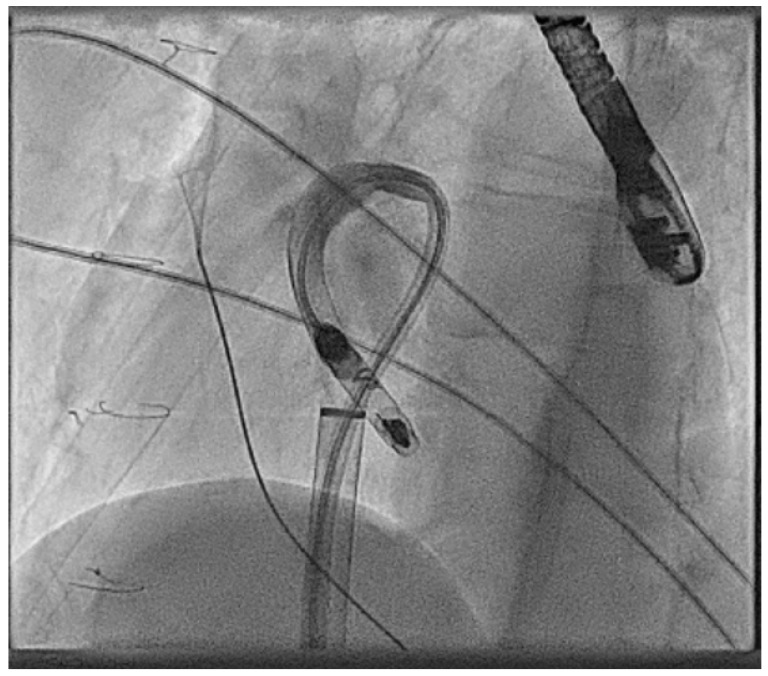
Extreme torque of the delivery catheter in an adult patient with an ECC to deliver the ventricular LPM.

Then, the delivery sheath must be carefully advanced over a stiff 0.035 support wire which can be difficult, and we normally try to make our puncture relatively vertical to make the passage easier. We would, at this point, administer heparin, aiming for a target activated clotting time of 300 or more similar to any left atrial-based procedure. From here, often a large amount of torque is needed to twist the delivery catheter to aim down into the ventricle and deliver the LPM (Figure 14).

In these cases, given the access difficulties, we would tend to accept any position with secure pacing and good parameters and would always check with transoesophageal echocardiography for any AV valve impingement.

**Figure 15:**
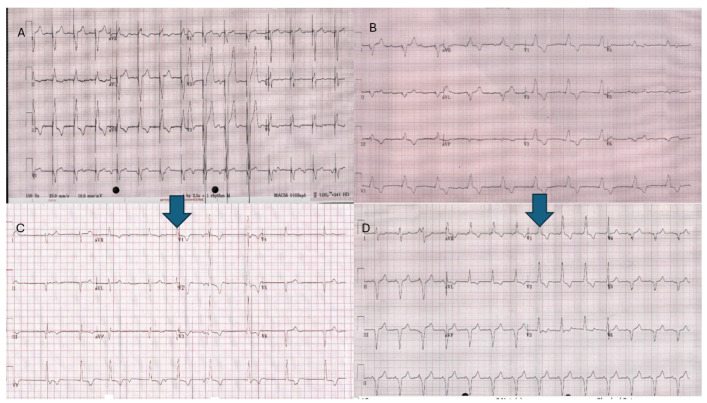
ECGs from two adult Fontan patients with epicardial pacing (**A**,**B**) and then leadless pacing (**C**,**D**) showing QRS narrowing.

The medium-term results (up to 3 years) for these cases have been acceptable so far with low rates of complications and good pacing results [59]. In some cases, we have been able to significantly improve QRS duration compared to the historical epicardial system using direct leadless pacing (Figure 15).

## 3. Conclusions

Leadless pacing has developed rapidly in recent years and now offers many options to safely overcome even the most complex CHD anatomy. In the coming years, further developments are expected with leadless conduction system pacing [32], totally wireless resynchronisation [60] and more co-implant procedures.

In addition to the newer technology and techniques, we hope that time will also increase the amount of data available to support LPMs in complex CHD.

## Figures and Tables

**Table 1:** A summary of the quoted technical specifications of current leadless pacing systems.

	Micra VR	Micra AV	Aveir AR	Aveir VR	Empower	EBRWISE
Length (mm)	25.9	25.9	32	38	31.9	9.1
Weight (g)	2	2	2.1	2.4	2	1.26
Width (mm)	6.7	6.7	6.5	6.5	6	2.7
Fixation	Passive tine	Passive tine	Active helix	Active helix	Passive tine	Active spike
Sensor	Accelerometer	Accelerometer	Temperature	Temperature	Accelerometer	N/A
Delivery catheter French size	23	23	25	25	21	12
Maximum mode	VVIR	VDD	AAIR	VVIR	VVIR	BIVP
Co-implant	No	No	Can be DR	Can be DR	With S-ICD	Mandatory
Designed to retrieve	No	No	Yes	Yes	Yes	No
Battery projected maximum	16 yrs	16 yrs	10 yrs	20 yrs	10 yrs	4.5 yrs

## Data Availability

No new data were created or analyzed in this study.

## Supplementary Materials

The following are available online at https://www.mdpi.com/article/10.3390/jcm14238560/s1, Video S1: Retrieval of a Micra™ over a tricuspid valve replacement using a dedicated retrieval sheath. Video S2: Direct uncovering of the delivery sheath in the right ventricle using a stiff wire for support in the pulmonary artery. Video S3: Dilatation of the Fontan baffle puncture with a non-compliant balloon showing the waist cracking.
